# Relationship between Serum Protein Electrophoresis, Endoscopic and Histopathological Scores in 99 Cats with Chronic Enteropathy

**DOI:** 10.3390/vetsci9090453

**Published:** 2022-08-24

**Authors:** Alessio Pierini, Eleonora Gori, Fiorenza Tulone, Elena Benvenuti, Enrico Bottero, Pietro Ruggiero, Veronica Marchetti

**Affiliations:** 1 Department of Veterinary Science, University of Pisa, Via livornese, San Piero a Grado, 56122 Pisa, Italy; 2 Professional Association Endovet, 00149 Rome, Italy

**Keywords:** electrophoresis, cats, chronic enteropathy, endoscopy, gamma-globulins

## Abstract

**Simple Summary:**

Serum protein electrophoresis (SPE) is a laboratory test used to separate different protein fractions. It is used to investigate animals with hyperglobulinemia and to distinguish between monoclonal and polyclonal gammopathies. SPE can also highlight acute or chronic inflammation patterns which are useful in disease monitoring. SPE is effective in humans with intestinal bowel disease (IBD) and chronic liver diseases. In addition, hypergammaglobulinemia is a marker of extraintestinal manifestations in human IBD patients, which often includes hepatic and pancreatic disease. Concurrent pancreatic and/or hepatic diseases in cats with chronic enteropathy (CE) have often been found, which may contribute to SPE alterations. The present study investigated the relationship between SPE and endoscopy, histopathology, and hepatic and pancreatic ultrasonographic findings in ninety-nine cats diagnosed with CE. No significant differences were observed between SPE and endoscopic and histopathological severity scores. Cats with concurrent pancreatic and/or hepatic ultrasonographic alterations showed lower albumin, lower α-globulin, and higher γ-globulin levels than cats diagnosed with enteropathy alone. This study suggests that hepatic and/or pancreatic alterations may influence SPE fractions in cats with CE, and should form the basis for further prospective studies on cats with diaditis and triaditis.

**Abstract:**

Few studies have investigated total protein (TP) and serum protein electrophoresis (SPE) in cats with chronic enteropathy (CE). Cats diagnosed with CE were evaluated to investigate the relationships between TP, SPE and endoscopy, histopathology, and extraintestinal involvement. Medical records were searched for cats with a history of chronic gastrointestinal signs and a final diagnosis of CE. Information on signalment, TP, SPE, endoscopic score, histopathological diagnosis and score, and concurrent hepatic or pancreatic ultrasonographic alterations was collected. Relationships between protein profiles and other variables were investigated. Ninety-nine cats were included in the study, 63 diagnosed with various degrees of bowel inflammation and 36 with small-cell alimentary lymphoma. The most common TP alteration was hypoproteinemia (24%). No significant differences were observed between protein profiles and endoscopic and histopathological severity scores. Forty-five cats showing concurrent pancreatic and/or hepatic ultrasonographic alterations, had significantly lower albumin, lower α-globulin, and higher γ-globulin levels than cats not showing concurrent alterations. Disease severity scores did not seem to influence the protein profile in cats with CE. Extraintestinal involvement may be suspected in cats with lower albumin and α-globulins and higher γ-globulins.

## 1. Introduction

Feline chronic enteropathy (CE) is an increasingly common disorder in elderly cats [1,2]. This disorder is defined as the presence of chronic (>3 weeks) gastrointestinal clinical signs in the absence of extraintestinal causes or infectious, obstructive, or localized neoplastic intestinal disease [3]. Although significant protein loss and the development of protein losing enteropathy is very rare in cats, CE-associated hypoalbuminemia has been reported in from 5 to 83% of cases [4,5,6,7,8,9,10]. In contrast, total protein (TP) is often normal or increased due to concurrent hyperglobulinemia and an increased TP concentration is part of the Feline Chronic Enteropathy Activity Index (FCEAI) [11].

Serum protein electrophoresis (SPE) is a laboratory test used to separate different protein fractions. It is used to investigate animals with hyperglobulinemia and to distinguish between monoclonal and polyclonal gammopathies [12].

In humans, SPE has been underused, but is promising in patients with intestinal bowel disease (IBD) and chronic liver diseases [13]. In addition, hypergammaglobulinemia in human IBD patients has been recently identified as a marker of extraintestinal manifestations [14,15] which often includes hepatic and pancreatic disease [16]. Concurrent pancreatic and/or hepatic diseases in cats with CE have often been found [17], which may contribute to TP and protein fraction alterations [18,19,20].

To the best of our knowledge, no studies have evaluated SPE in cats with CE. We hypothesized that TP and SPE fraction levels may vary in relation to different histopathological and endoscopic severity scores of feline CE and the presence of concurrent hepatic and/or pancreatic disease. The aim of this study was to investigate the relationship between TP and SPE with endoscopic and histopathological scores in cats with CE. The possible relationship between SPE alterations and concurrent hepatic and/or pancreatic ultrasonographic alterations was also investigated.

## 2. Materials and Methods

The medical records of cats referred to the Veterinary Teaching Hospital (VTH) of the University of Pisa from 2014 to 2019 were retrospectively searched for cats with chronic enteropathy. For inclusion in the study, cats had to meet all the following criteria: (1) a history of chronic (>3 weeks) gastrointestinal signs (at least one of the following: vomiting, diarrhea, or weight loss); (2) exclusion of primary extraintestinal disease (e.g., renal failure assumed as creatinine values above the upper interval range, endocrinopathy, tumor), anatomic intestinal disease (e.g., intussusception, foreign body), intestinal parasites using blood analysis, urinalysis, fecal flotation and imaging performed within a month before endoscopy (3) failure to respond to dietary therapy or to probiotics and prebiotics; (4) gastrointestinal endoscopy biopsies and histopathological diagnosis of intestinal inflammation or small cell alimentary lymphoma (SCAL); and (5) an SPE performed on serum collected at the time of endoscopy. Cats that failed to meet the inclusion criteria and cats affected by FIV or FeLV were excluded from the study. Cats with intermediate or large cell alimentary lymphoma were excluded. Cats with suspected or documented exocrine pancreatic insufficiency, hepatic failure, extrahepatic biliary obstruction, and pancreatic, or hepatic tumors were also excluded. Cats with suspected pancreatic or hepatic inflammatory disease were included for the aim of the study. No cats had a recent history of treatment with immunomodulatory drugs; however other drugs (e.g., antibiotics, antiacids, non-steroidal anti-inflammatory drugs) were allowed.

Data on clinical history, signalment, complete blood count, serum biochemistry, total thyroxine concentration, fecal flotation, urinalysis, and abdominal ultrasound were retrieved from medical records.

Each cat had TP (Liasys—Assel SRL, Rome, Italy) and agarose gel SPE (Interlab Pretty platform—Interlab S.R.L., Rome, Italy) measured at our Laboratory of Clinical Pathology of the VTH. Routinely, SPE is performed on split beta agarose gel using an automated platform and according to the manufacturer’s instructions, with a required sample volume of 30 μL. The laboratory performs electrophoresis employing a specific kit (SRE602K, Interlab S.R.L., Rome, Italy). SPE results are shown using the manufacturer’s software and each fraction is separated using a standardized method into four distinct and well-defined zones or bands: albumin, total alpha (α), total beta (β), and gamma (γ) globulins. The SPE machine is connected to a computer that analyzes the data and produces a densitometer trace, showing protein fractions and reference ranges (internal laboratory reference ranges calculated for cats). In this study, we considered TP (g/dL) and both absolute and percentage values of albumin, α-globulins, β-globulins, and γ-globulins. Each SPE fraction was considered decreased or increase if their respective absolute and/or percentage value was below or above the reference range.

Endoscopic reports of each cat were retrospectively reviewed. The endoscopic evaluation was performed following WSAVA guidelines [21]: a severity score from 0 to 3 was assigned to characterize each tract as normal (0), or with mild (1), moderate (2), or severe (3) lesions. Histopathological reports of intestinal biopsies were also reviewed. In accordance with WSAVA guidelines, at least eight biopsies from each gastrointestinal region are usually obtained. Sample size, orientation, and quality were routinely evaluated for the histopathological examination by the pathologists, however, the number of total biopsies submitted to the pathologist and the number of adequate samples were not reviewed. Cats were excluded if endoscopic biopsies were considered inadequate to reach a diagnosis. Biopsies were evaluated by different pathologists and scored following the WSAVA guidelines into four categories of intestinal inflammation severity: normal, mild, moderate, and marked inflammation [22]. Although differentiating SCAL from IBD can be extremely difficult in cats using histomorphology alone [23,24], in this study cats were diagnosed with SCAL based on the pathologists’ sample interpretations. Due to the retrospective nature of the study further investigations, such as immunohistochemistry (IHC) or PCR for antigen receptor rearrangements (PARR), were not possible.

Based on the endoscopic score, cats were divided into two groups: Group Endo1 (cats with an endoscopic score of 1) and Group Endo2-3 (cats with an endoscopic score of 2 or 3). No cats had an endoscopic score of 0. Based on the histopathological score, cats were thus divided into two groups: HistoA (cats with mild or moderate CE) and HistoB (cats with severe inflammation or SCAL). To prevent misclassification of the endoscopic and histopathological groups, the highest score (i.e., the most severe) of all gastrointestinal segments was used.

Only cats with an abdominal ultrasound performed within two weeks from the endoscopy and with reports available were included in order to investigate the relationship between TP and SPE and concurrent hepatic and/or pancreatic ultrasonographic alterations. Based on ultrasonographic findings, cats were divided into two groups: Group UI (ultrasound with intestinal findings) and Group UEI (ultrasound with extra-intestinal findings). Cats were assigned to group UI if no ultrasonographic alterations of the liver or pancreas were reported, and to group UEI if at least two alterations compatible with hepatopathy or pancreatopathy were reported. Changes in liver echogenicity, parenchymal size, homogeneity, presence of thickened gallbladder wall, biliary sludge. And dilatation or thickness of the bile duct were considered compatible with hepatopathy [25,26]. Irregular pancreatic contour, changes in parenchymal echogenicity, increased pancreatic duct size, and peripancreatic free fluid or hyperechoic mesentery were considered compatible with pancreatopathy [27].

Descriptive data were used to define population characteristics. Continuous data were tested for normality using the Kolmogorov-Smirnov test. Variables were then reported as median and range as they were non-normally distributed. Continuous data between Endo1 vs. Endo2-3, HistoA vs. HistoB, and I vs. EI were compared using the Mann-Whitney U-test for unpaired samples. Statistical software was used for data analysis (IBM SPSS Statistics, v. 25, IBM Corporation, Armonk, NY, USA; GraphPad Prism 7 for Mac OS) and significance was set at *p* < 0.05.

## 3. Results

Ninety-nine cats met the inclusion criteria and were included in the study. Fifty-three cats were neutered males, 43 were spayed females and three were intact females. Breeds included European domestic shorthair (*n* = 92), Chartreux (*n* = 2), Bengal (*n* = 1), British shorthair (*n* = 1), Persian (*n* = 1), Maine Coon (*n* = 1), Siberian (*n* = 1). Age ranged from 1 to 17 years (median 10 years).

Seventy cats presented vomiting, 67 has weight loss, and 37 had diarrhea. Forty-eight cats had concurrent weight loss and vomiting, and 29 had concurrent weight loss and diarrhea. Eleven cats showed weight loss, diarrhea, and vomiting simultaneously.

**Figure 1:**
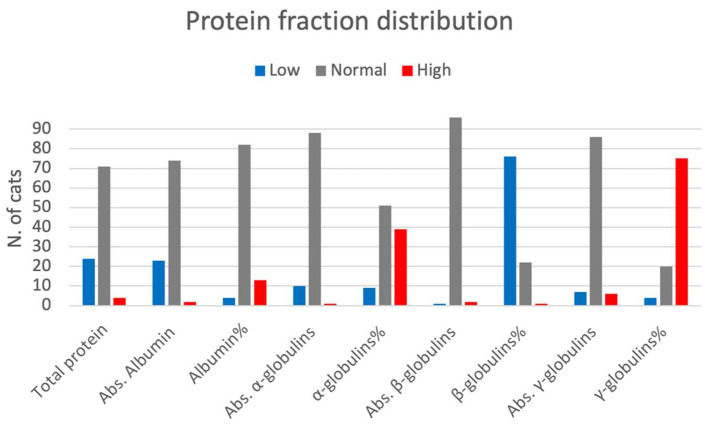
The figure shows the distribution of protein fractions in cats (descriptive statistics). Abs: absolute values. Reference ranges: Total protein 5.5–8 g/dL; Abs. Albumin (2.8–4.0 g/dL); Albumin% (41.6–56.5%); Abs. α-globulins (0.8–2.1 g/dL); α-globulins% (13.2–22.4%); Abs. β-globulins (0.3–1.5 g/dL); β-globulins% (13.1–27.7%); Abs. γ-globulins (0.4–2.0 g/dL); γ-globulins% (6.6–11.6%).

The median TP was 6.0 g/dL (range 3.4–10.1). Seventy-one cats had TP values within the reference range (RR, 5.5–7.8 g/dL), 24 cats had protein levels lower than RR and four had protein levels higher than RR. No cats showed monoclonal gammopathy. Protein fraction distributions are shown in Figure 1.

Gastric and duodenal biopsies were collected in all cats, while ileal and colonic biopsies were collected in 13 and 26 cats, respectively. Forty-six cats were included in Endo1 (mild), and 53 cats were classified as moderate (*n* = 42) or severe (*n* = 11) and were included in Endo2-3. No significant differences in SPE and TP were observed between the two Endo groups (Table 1).

Regarding the histopathological evaluation, 42 cats showed mild (*n* = 16) or moderate (*n* = 26) inflammation and were included in HistoA, whereas 57 cats had marked inflammation (*n* = 21) or SCAL (*n* = 36) (HistoB). Marked inflammation was observed in biopsies: 5 gastric, 14 duodenal, 1 ileal, and 1 colonic. Thirty SCALs were diagnosed from duodenal biopsies, and six from ileum biopsies. No significant differences were observed in SPE and TP between the two Histo groups (Table 1).

A total of 75 of the 99 cats had an abdominal ultrasound performed within two weeks from endoscopy and with reports available for review. Thirty cats were included in group UI and 45 in group UEI. In the UEI group, 22 had hepatic changes, 5 had pancreatic changes and 18 had both. Group UEI showed lower serum albumins, lower α-globulins, and higher γ-globulins than group UI (Table 1).

## 4. Discussion

In human gastroenterology, SPE has been suggested as an indicator of IBD activity and to monitor patient remission [13]. In cats, SPE is commonly used to support the diagnosis of infectious or neoplastic diseases [28,29,30,31]. In our study, SPE was evaluated in cats with CE to investigate the possible relationship between protein fractions and endoscopy and histological severity scores, and concurrent hepatic and/or pancreatic ultrasonographic alterations.

Since the clinical presentation, laboratory tests, endoscopic, and histopathological findings can overlap in severe IBD and SCAL [23,24], we decided to include them in the same group.

In our study, hypoproteinemia was a common finding and was observed in 24% of cats while hypoalbuminemia occurred only in 5% of cases. Hypoproteinemia is quite variable in cats with IBD or gastrointestinal lymphoma, occurring in 21–83% of cases [6,9,10]. The decrease in TP in our population could have a multifactorial etiology: intestinal loss of proteins (both albumin and globulin), reduced albumin and/or globulin synthesis during inflammation or chronic hepatitis [10,30,32]. The protein loss throughout the intestinal wall is less common in cats than in dogs, and, similarly to our finding, hypoalbuminemia has been infrequently reported in cats with CE [6,9,33,34]. Moreover, the present study was not able to demonstrate any significant difference in TP and SPE fractions between endoscopic and histological groups.

The differences in albumin, α- and γ-globulins in cats with and without concurrent sonographic extraintestinal organ changes observed in this study are interesting. Group UEI showed lower albumin levels than cats in group UI. Different mechanisms could explain the lower albumin levels: larger tissue inflammation, decrease hepatic synthesis, and chronic malnutrition [30]. Extraintestinal involvement may mean larger tissue damage and inflammation with a subsequent proportional decrease in albumin, as expected by a negative acute phase protein. Cats with CE and concurrent increase in feline pancreatic lipase immunoreactivity (fPLI) have been reported to have lower albumin levels than cats with normal fPLI [18]. Moreover, cats with moderate to severe pancreatitis are significantly more likely to have hypoalbuminemia than healthy cats and cats with mild pancreatitis [35]. Otherwise, hepatic involvement can lead to a reduced hepatic function with a decrease in albumin synthesis ability [30]. Although hypoalbuminemia does not seem prognostic in cats with IBD and concurrent pancreatitis, the detection of hypoalbuminemia may help clinicians to hypothesize concurrent liver and/or pancreas involvement.

The reduction in α-globulins in cats with UEI was an unexpected finding. We hypothesized that greater tissue inflammation and damage as suspected in group UEI would be associated with an increase in positive acute phase proteins (e.g., serum amyloid A) which usually migrate in this fraction [36,37]. However similarly to albumin, chronic malnutrition, and impaired liver function may reduce α-globulins [10,32,33]. A reduction in daily diet intake in cats with the gastrointestinal disease is frequently encountered. Fasting combined with stress increases peripheral lipolysis leading to lipid mobilization and intracellular accumulation, with reduced export [38]. It has been suggested that concurrent protein deficiency and negative nitrogen balance associated with fasting reduce the ability to produce apoproteins to export fat from the liver and that taurine and carnitine deficiencies contribute to the pathogenesis [38]. In the authors’ opinion, intracellular accumulation of lipids may interfere with the metabolic activity of the cells with a reduction of hepatic protein synthesis, which can turn into a vicious circle of malnutrition [38]. However, in the present study, no information on body condition and muscle mass scores and weight loss was collected and analyzed making any further considerations challenging.

Elevated γ-globulin levels are associated with chronic antigenic stimulation, as this fraction is composed of immunoglobulins, particularly IgG. A wide variety of diseases increase γ-globulins, with immune-mediated and infectious diseases being the most likely cause found in a study on 155 cats [31]. Although γ-globulins did not differ between the endoscopy and histopathological severity scores in the present study, cats diagnosed with concurrent sonographic extraintestinal organ changes showed higher γ-globulins than cats without sonographic changes. In pediatric inflammatory bowel disease patients, elevated γ-globulins have been associated with extra-intestinal manifestations [14]. The pathogenesis of extra-intestinal manifestations in IBD in people is still unknown, although it is presumed that immune responses in extraintestinal sites can be triggered by intestinal barrier injury [16]. Similarly, an immune-mediated pathogenesis has been hypothesized in cats with CE and concurrent hepatic and pancreatic disease [17]. Elevated γ-globulins could therefore suggest hepato-pancreatic involvement in cats with CE.

Interestingly, the evaluation of SPE percentage values showed more frequent SPE fraction alterations than the evaluation of absolute values. However, the statistical analysis of absolute or percentage values produced the same results. The statistical analysis of qualitative variables (hypo-, normo- or hyper-SPE fraction) showed no association with any of the groups analyzed in the present study (data not shown). In our opinion, the reference ranges of SPE in cats are not well-established which might lead to a misclassification of cats in terms of qualitative variables.

The present study has some limitations. Due to its retrospective nature, standardization of the endoscopy and histopathological evaluation was not possible. Endoscopy and histopathology were performed by several endoscopists and pathologists, respectively, following WSAVA recommendations. However, as endoscopic images and sample slides were not always reviewed by the same investigator, operator biases cannot be excluded. Extraintestinal involvement has been assigned based on ultrasonography findings [39]. The collection of clinicopathological findings including liver enzymes, and fPLI would have resulted in a more accurate classification of cats, especially in terms of pancreatopathy [27]. However, it has been reported that ultrasonography findings and clinicopathological abnormalities are less able to correctly classify intestinal, hepatic, or pancreatic inflammation in cats with gastrointestinal signs when compared with tissue biopsy and histopathological evaluation [40].

## 5. Conclusions

This study confirms that hypoproteinemia and hypoalbuminemia are infrequently observed in cats with CE and that albumin and SPE fraction levels are not correlated with the severity of the disease. We also found interesting differences in SPE fractions in cats with concurrent pancreatic or hepatic ultrasonographic alterations. However, the present findings should be interpreted with caution since the lack of liver and pancreas histopathological investigation.

## Figures and Tables

**Table 1:** Comparisons of SPE between groups based on endoscopy, histopathology and ultrasonographic findings.

	Endoscopy			Histopathology			Ultrasonographic Findings		
	Endo1 *n* = 46	Endo2-3 *n* = 53	*p*	HistoA *n* = 42	HistoB *n* = 57	*p*	UEI *n* = 45	UI *n* = 30	*p*
Total protein	6 (4–8.5)	6 (3.4–10.1)	0.378	6 (3.4–9.9)	6.1 (4.2–10.1)	0.202	7.1 (4.7–10.1)	7.2 (5.1–10)	0.897
Absolute values (mg/dL)									
Albumin	3 (2.1–3.6)	3 (1.9–4.6)	0.444	3 (1.9–3.9)	3.1 (2.2–4.6)	0.090	3.3 (1.6–4.6)	3.5 (2.3–4.5)	0.074
α-globulins	1.2 (0.5–2)	1.3 (0.4–2.5)	0.138	1.3 (0.5–2)	1.3 (0.4–2.5)	0.579	1.1 (0.6–2.5)	1.6 (0.4–2)	**0.009**
β-globulins	0.6 (0.2–2)	0.6 (0.3–1.7)	0.420	0.6 (0.2–1.7)	0.7 (0.3–2)	0.063	0.9 (0.3–2)	0.8 (0.3–1.9)	0.295
γ-globulins	1 (0.2–2.2)	1 (0.3–5.4)	0.872	0.9 (0.2–5.4)	1 (0.2–2.9)	0.294	1.5 (0.3–5.4)	1.2 (0.4–2.9)	**0.034**
Percentage (%)									
Albumin	50.5 (34.8–69.1)	50.2 (26.8–63.1)	0.861	50.3 (26.8–69.1)	50.3 (34.8–61.6)	0.599	45.9 (26.8–61.9)	49.5 (37.9–62.1)	**0.047**
α-globulins	20.6 (9.1–31.2)	21.8 (6.9–33.8)	0.260	21.5 (9.1–31.2)	20.6 (6.9–33.8)	0.496	17.5 (7.8–29.3)	21 (6.9–33.8)	**0.02**
β-globulins	10.4 (6–23.7)	10.1 (4.6–30.6)	0.154	9.4 (4.6–30.6)	11.5 (5.3–23.7)	0.182	14.2 (5.1–26.1)	11.4 (4.6–27.4)	0.100
γ-globulins	17.4 (5.2–30.1)	16.4 (4.6–54.9)	0.869	16.1 (5.2–54.9)	17.4 (4.6–30.1)	0.414	20.4 (6.1–54.9)	16.8 (5.9-33.8)	**0.009**

Endo1: cats with endoscopic score 1; Endo2-3 included cats with endoscopic score 2 or 3. HistoA included cats with histological classification of mild or moderate inflammation; HistoB included cats with histological classification of severe inflammation or small cell alimentary lymphoma. UEI included cats with concurrent ultrasonographic hepatic or pancreatic changes; UI included cats with no ultrasonographic hepatic or pancreatic changes. Significances are reported in bold.

## Data Availability

Not applicable.
